# Association between dietary antioxidant indices and glaucoma in the National Health and Nutrition Examination Survey

**DOI:** 10.3389/fnut.2023.1304809

**Published:** 2023-11-22

**Authors:** Wenwei Li, Bin Wang

**Affiliations:** Department of Ophthalmology, Tongde Hospital of Zhejiang Province, Hangzhou, Zhejiang Province, China

**Keywords:** dietary, glaucoma, antioxidant, NHANES, nutrition epidemiology, cross-sectional study

## Abstract

**Objective:**

To explore the relationship between dietary antioxidant indices (DAI) and glaucoma using the data from the 2005 to 2008 National Health and Nutrition Examination Survey (NHANES).

**Methods:**

Our study comprised participants who completed the NHANES dietary intake interview and visual health questionnaire at age 40 or older. The intakes of the vitamins A, C, and E as well as of zinc, selenium, and magnesium were used to generate the DAI, which represents the overall antioxidant qualities. The self-report method for glaucoma diagnosis (ever been told by an eye doctor) was used. Survey logistic regression analyses were employed to investigate the association between DAI and glaucoma.

**Results:**

A total of 6,128 participants were included in our study. The unadjusted model’s findings revealed a negative correlation between dietary antioxidant indices and self-reported glaucoma [0.93 (0.90, 0.96), *p* < 0.0001]. For every unit increase in dietary antioxidant indices, the risks of self-reported glaucoma in model 1 (adjusted for age, sex, race, marital status and PIR) decreased by 5% [0.95 (0.90, 0.99), *p* = 0.02]. After adjusting all the covariates (model 2), the risks of self-reported glaucoma decreased by 6% [0.94 (0.90, 0.99), *p* = 0.02] for each unit increase of dietary antioxidant indices. After converting DAI into classified variables (tertile), the same trend was found (*p* = 0.001).

**Conclusion:**

In this analysis of the NHANES database, we found higher dietary antioxidant indices were associated with lower risk of glaucoma.

## 1 Introduction

Glaucoma is a set of ophthalmological disease, which results in deteriorative impairment to the optic nerve. It is believed to be the second leading cause of irretrievable blindness globally, and it is anticipated that 111.8 million people will suffer from glaucoma in 2040 (1). Patients often do not have any typical symptoms until to the late phase when they suffer from server vision loss problems. It is estimated that about half patients with glaucoma do not get correct diagnosis even in the developed countries (2, 3). Unlike cataract, this vision loss cannot be recovered by any medications or surgical treatments. Data has shown that giant global burdens have been brought by glaucoma around the world.

According to definition, oxidative stress is believed to be the imbalance between prooxidants and antioxidants (4). Although at appropriate concentrations it is essential in varieties of physiological responses in cells including cell proliferation, apoptosis, and gene expression, it is still considered as a risk factor for a lot of diseases (5). Antioxidants are believed to be some molecules which can slow down or avoid the process of oxidizing of other molecules. At the same time, antioxidant, being able to remove free radicals as well as prevent lipid peroxidation, has caught wide attention during the last decade. Many types of chronic diseases have been demonstrated to have a close relation with the imbalance between oxidant and antioxidant (6, 7). Some studies have suggested that oxidative stress has taken part in the pathology development of glaucoma (8–11). In theory, antioxidants may have a positive effect on patients suffering from glaucoma. However, results from these studies are not conclusive. With these conclusions from the above studies, we can confer that oral antioxidant substances may have some beneficial effect on glaucoma.

As far as we are aware, although there existed some studies involving the association between antioxidants and glaucoma, the results were not consistent. No definite association between antioxidants and glaucoma has been verified. Therefore, the purpose of our study is to examine the association between dietary antioxidants indices (DAI) and glaucoma in the 2005–2008 National Health and Nutrition Examination Survey (NHANES) population.

## 2 Materials and methods

### 2.1 Sample and population

The 2005–2008 NHANES database was obtained for subsequent analysis. Created in the 1960s by the Centers for Disease Control and Prevention, NHANES was organized to evaluate the health status of US adults and children. By applying stratified multistage sampling design, about 10,000 volunteers take part in the NHANES to take blood tests, interviews, and physical examinations every year. And then, data from these surveys are composed to produce weighted estimates, which can be regarded as a representative of the US population. We use the 2005–2008 NHANES database to assess the association between dietary antioxidant indices and self-reported glaucoma. Since all participants provide written informed consent prior to completing the NHANES and these data were available publicly and de-identified, it was considered exempt from Committee for Human Research approval.

### 2.2 Measures

Dietary information was collected by using the 2-day 24-h dietary recall by proficient interviewers from the Centers for Disease Control and Prevention. Participants were invited to the Mobile Examination Center and then asked about the details of the dietary intake during the 24-h period prior to the interview. After 3 to 10 days, the second interview is applied by telephone enquiry. Six dietary antioxidant micronutrients, including zinc, magnesium, selenium, vitamins A, C, and E, were utilized to compute the DAI by using the approach suggested by Wright et al. in the previous study (12). To calculate the DAI, we normalized each of the abovementioned antioxidant micronutrients by taking the mean out and dividing by the standard deviation (SD). The composite DAI is then determined by weighted addition of the standardized intakes of each of these micronutrients.

The interviewer asked the participants whether they had glaucoma or not by using the following question: “Ever told had glaucoma by an eye doctor?.” The answer responding to this question was “Yes,” “No,” “Do not know” and “Missing.” The answers with “Do not know” and “Missing” were deleted for further research.

The inclusion criteria are as follows: those whose DAI can be obtained and calculated; those who have clear answers to ever told had glaucoma. The exclusion criteria are those who can not provide DAI by calculating relative micronutrients or those who can not offer glaucoma information.

### 2.3 Covariates assessment

Other potential confounding variables in this study included age, sex, ethnicity, marriage, poverty income ratio (PIR), body mass index (BMI), educational level, smoke, hypertension, hyperlipidemia, alcohol, chronic kidney disease (CKD), and diabetes (DM).

### 2.4 Statistical analysis

Mean and SD were applied to describe continuous variables when it is normally distributed, otherwise median values and interquartile ranges were used instead. When it is normally distributed, t test was used to compare between groups; if not, the Mann–Whitney test was applied. Percentages was utilized to describe categorical variables andχ2 test was applied to compare between groups. Multivariable logistic regression was applied to assess the association between dietary antioxidant indices and self-reported glaucoma. Crude model was applied to solely check the relationship between these two variabilities. Model 1 investigated the relationships with the adjustment of age, sex, race, marriage and PIR. The final model was adjusted for age, sex, ethnicity, marriage, PIR, BMI, educational level, smoke, hypertension, hyperlipidemia, CKD and diabetes. To confirm the relationship, we also converted DAI into classified variables for analysis using the quartile method. And the value of p of the trend was computed to detect the possibility of nonlinearity.

All statistical analyses were conducted in R version 4.1.2 with appropriate sampling weights to account for the complex survey design. All *p* values with *p* < 0.05 were believed to be statistically significant.

## 3 Results

### 3.1 Characteristics of participants included

After following the inclusion and exclusion criteria, there were 6,128 participants included, aged 40 years or older, who reported whether they had been diagnosed as glaucoma or not in the 2005–2008 NHANES cycles. These participants were evaluated to represent 115.02 million US residents. Demographics along with basic health characteristics of these participants were presented in Table 1. The participants who had been told to be diagnosed with glaucoma were about 10 years older than those who had not. Participants with self-reported glaucoma were more inclined to have a lower PIR [2.90 (0.10) vs. 3.28 (0.07), *p* = 0.001], to be more with black [16.01 (11.26, 20.75) vs. 9.70 (7.18, 12.22), *p* = 0.01], to be more with widowed [22.68 (18.72, 26.64) vs. 8.73 [7.68, 9.78], *p* < 0.0001], to be less with college or superior degree [19.36 (14.35, 24.36) vs. 31.32 [28.01, 34.64], *p* < 0.001], to be more with hypertension [68.54 (62.18, 74.90) vs. 49.20 (46.87, 51.52), *p* < 0.0001], to be less with never smoke [43.63 (37.70, 49.55) vs. 49.31 (46.86, 51.76), *p* < 0.001], to be more with CKD [82.74 (77.71, 87.77) vs. 78.57 (76.91, 80.23), *p* < 0.0001], to be more with DM [29.68 (24.62, 34.74) vs. 17.01 (15.38, 18.63), *p* < 0.0001]. Differences in BMI, sex, hyperlipidemia, and alcohol use were not statistically significantly different between these two groups.

**Table 1:** Baseline characteristics of participants included with and without glaucoma from 2005–2008 NHANES cycle.

Characteristic	No self-reported glaucoma	Self-reported glaucoma	*p* value
Age, mean (SD)(years)	56.57 (0.40)	66.45 (1.07)	<0.0001
PIR, mean (SD)	3.28 (0.07)	2.90 (0.10)	0.001
BMI, mean (SD)(kg/m2)	29.12 (0.14)	28.79 (0.33)	0.36
DAI	1.26 (0.11)	−0.01 (0.27)	<0.0001
Sex (%)			0.68
Female	53.72 (52.13, 55.30)	52.32 (46.23, 58.42)	
Male	46.28 (44.70, 47.87)	47.68 (41.58, 53.77)	
Race (%)			0.01
White	77.71 (73.97, 81.46)	73.81 (66.86, 80.76)	
Black	9.70(7.18, 12.22)	16.01 (11.26, 20.75)	
Mexican	5.25 (3.94, 6.57)	3.70 (1.33, 6.07)	
Other Race	7.33 (5.72, 8.94)	6.49 (2.67, 10.31)	
Marriage (%)			<0.0001
Widowed	8.73 (7.68, 9.78)	22.68 (18.72, 26.64)	
Married	65.37 (62.74, 68.01)	54.14 (48.62, 59.67)	
Never married	6.00 (4.94, 7.07)	5.16 (2.05, 8.26)	
Divorced	13.29 (11.74, 14.84)	11.64(8.51, 14.77)	
Separated	2.41 (1.78, 3.04)	3.30 (0.99, 5.62)	
Living with partner	4.19 (3.33, 5.06)	3.07 (0.57, 5.57)	
Education (%)			<0.001
Some College or AA degree	31.83 (29.29, 34.36)	33.71 (26.28, 41.15)	
College Graduate or above	31.32 (28.01, 34.64)	19.36 (14.35, 24.36)	
High School Grad/GED or Equivalent	29.42 (26.88, 31.96)	32.61 (25.55, 39.66)	
Less Than 9th Grade	7.43 (5.96, 8.90)	14.33 (8.48, 20.17)	
Hypertension (%)			<0.0001
No	50.80 (48.48, 53.13)	31.46 (25.10, 37.82)	
Yes	49.20 (46.87, 51.52)	68.54 (62.18, 74.90)	
Smoke (%)			<0.001
Never	49.31 (46.86, 51.76)	43.63 (37.70, 49.55)	
Former	30.80 (28.95, 32.66)	43.84 (39.09, 48.60)	
Now	19.88 (17.70, 22.07)	12.53(7.64, 17.42)	
CKD (%)			<0.0001
No	81.37 (79.58, 83.15)	63.70 (56.65, 70.75)	
Yes	18.63 (16.85, 20.42)	36.30 (29.25, 43.35)	
Hyperlipidemia (%)			0.14
No	21.43 (19.77, 23.09)	17.26 (12.23, 22.29)	
Yes	78.57 (76.91, 80.23)	82.74 (77.71, 87.77)	
DM (%)			<0.0001
No	72.27 (70.01, 74.54)	61.58 (56.68, 66.48)	
DM	17.01 (15.38, 18.63)	29.68 (24.62, 34.74)	
IFG	5.02 (4.27, 5.76)	3.74 (1.78, 5.69)	
IGT	5.70 (4.75, 6.66)	5.01 (2.65, 7.36)	
Alcohol user (%)			0.07
Never	11.62 (9.89, 13.36)	12.86 (8.83, 16.89)	
Former	21.90 (19.87, 23.93)	30.56 (24.51, 36.61)	
Mild	39.71 (37.19, 42.23)	34.13 (28.66, 39.59)	
Moderate	14.28 (13.02, 15.54)	12.16(5.77, 18.55)	
Heavy	12.49 (11.14, 13.83)	10.29(6.03, 14.55)	

SD, standard deviation; PIR, poverty-income ratio; BMI, body mass index; DAI: dietary antioxidant indices; CKD, chronic kidney disease; DM, diabetes mellitus; IFG, impaired fasting glucose; IGT, impaired glucose tolerance.

### 3.2 Multivariable analysis

Participants with 40 years or older who reported whether they had been told had glaucoma or not were included in this study for further analysis. And the participants met above standards all have the dietary data. Among these, 6.41% had been told had glaucoma.

**Figure 1:**
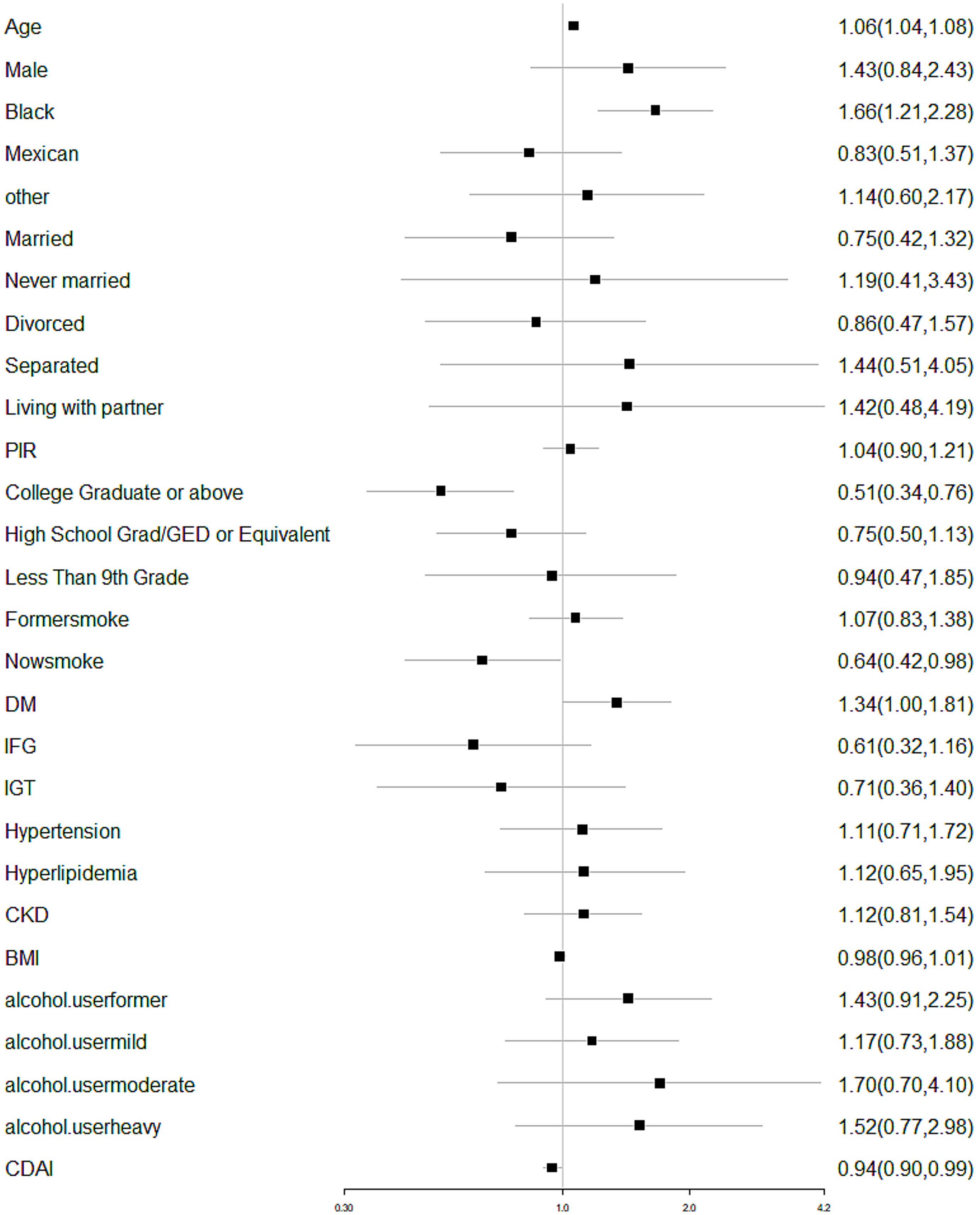
Forest plot showing the association between ADI and glaucoma. ROS, reactive oxygen species; PIR, poverty-income ratio; IOP, intraocular pressure; DM, diabetes mellitus; IFG, impaired fasting glucose; IGT, impaired glucose tolerance; CKD, chronic kidney disease; BMI, body mass index; CDAI: composite dietary antioxidant indices; TM, trabecular meshwork.

Multivariable logistic regression analysis was performed to analyze the association between dietary antioxidant indices and self-reported glaucoma, and a consistent association was found (Table 2). The unadjusted model’s findings (crude model) revealed a negative correlation between dietary antioxidant indices and self-reported glaucoma [0.93 (0.90, 0.96), *p* < 0.0001]. For every unit increase in dietary antioxidant indices, the risks of self-reported glaucoma in model 1 (adjusted for age, sex, race, marital status and PIR) decreased by 5% [0.95 (0.90, 0.99), *p* = 0.02]. After adjusting all the covariates (model 2), the risks of self-reported glaucoma decreased by 6% [0.94 (0.90, 0.99), *p* = 0.02] for each unit increase of dietary antioxidant indices (Figure 1). After converting DAI into classified variables (tertile), the same trend was found (*p* = 0.001). Besides, we also made a subgroup analysis according to the sex.

**Table 2:** Logistic regression models of CDAI for self-reported glaucoma status.

Variable	Crude Model		Model I		Model II	
	OR (95%CI)	*p*-value	OR (95%CI)	*p*-value	OR (95%CI)	*p*-value
DAI	0.93 (0.90, 0.96)	<0.0001	0.95 (0.90, 0.99)	0.02	0.94 (0.90, 0.99)	0.02
DAI(divided into 3 groups)						
Tertile 1	Ref		Ref		Ref	
Tertile 2	0.76 (0.58, 0.99)	0.05	0.73 (0.54, 1.00)	0.05	0.62 (0.44, 0.88)	0.01
Tertile 3	0.47 (0.33, 0.67)	<0.001	0.53 (0.33, 0.85)	0.01	0.47 (0.29, 0.78)	0.01
P for trend		0.001		0.001		0.001

OR, odds ratio; CI, confidence interval.

Crude Model adjust for: none; Model I adjust for: age, sex, race, marital status, PIR.; Model II adjust for: age, sex, race, marital status, PIR, CKD, BMI, education level, hyperlipidemia, diabetes, hypertension, smoking status, alcohol consumption.

## 4 Discussion

In this study, representing 115.02 million US residents with years of 40 or older, we found that with the increase of dietary antioxidant indices, the probabilities of self-report glaucoma decreased.

Different opinions appeared when analyzing the association between antioxidant activity and glaucoma in the literature. Abu-Amero et al. obtained blood from 139 glaucoma patients and 149 glaucoma-free controls. By using spectrophotometric and enzyme-linked immunosorbent assay methods, they found that there existed a statistically significant inverse correlation between total antioxidant concentration and intra ocular pressure. Hence, they suggested that total antioxidant level may be a potential role as a predictive-marker for glaucoma (13). Ferreira et al. studied total reactive antioxidant potential and antioxidant enzymes in aqueous humor, and they found that glaucoma patients presented with significantly decreased total reactive antioxidant potential and increased superoxide dismutase (14). In Sorkhabi’s study, during surgery, samples of aqueous humor and serum were taken from 27 patients with cataracts and 28 patients with glaucoma. Patients with primary open angle glaucoma had lower blood levels of total antioxidant status than controls with cataracts (15). Erdurmus et al. found that decreased antioxidant defense and increased oxidative stress system may play a large part in the development of primary open-angle glaucoma and pseudoexfoliative glaucoma by spectrophotometry (16). Nucci et al. studied 40 patients with primary open-angle glaucoma and 26 patients with cataract, and demonstrated that oxidative stress and decreased antioxidant defenses were involved in glaucoma (17). Takayanagi et al. also suggested that lower level of systemic antioxidant capacity was in close relation with glaucoma patients (18). Himori et al. suggested that individuals with low levels of systemic antioxidants may be more vulnerable to oxidative stress, which might quicken the onset of glaucoma (19).

However, Yuki et al. obtained blood sample from 43 normal-tension glaucoma patients and 40 glaucoma-free controls. They found that patients with normal-tension glaucoma presented with an increase level in the serum total antioxidant (20). Kang et al. studied the relation between dietary antioxidant intake and primary open-angle glaucoma risk by examining participants from the Nurses’ Health Study (n = 76,200) and the Health Professionals Follow-up Study (n = 40,284). No associations between antioxidant intake and the risk of primary open-angle glaucoma were observed (21). Their results differed with our study, which could be explained by the following several reasons. Firstly, the data in Kang’s study was a little older, which was from the 1980s or 1990s, while our study only included data from the 2005 to 2008. Secondly, the participants in their study were all from medical care related professions, either nurses, dentists, veterinarians, pharmacists, optometrists, osteopaths or podiatrists, restricting their conclusion to the limited people, while the participants from our study were not limited to these jobs. Ergan et al. made a analysis to evaluate total antioxidant status, total oxidant status, and the oxidative stress index of the aqueous humor in patients with glaucoma, and found that high levels of total antioxidant status were observed in patients with glaucoma (22).

It is understood that oxidative stress plays a significant role in the cell death process in neurodegenerative illnesses, especially in glaucoma. TM (trabecular meshwork) is believed to be vulnerable to oxidative stress (23). The TM’s endothelial cells, in particular, are affected by oxidative degenerative processes, which are connected to a rise in IOP (intraocular pressure). Clinically meaningful harm to TM cells’ lysosome system has been found after prolonged exposure to oxidative stress. The physiological degradation of TM tissue, which was demonstrated to be caused by phenotypic changes affecting the milieu of TM tissue, is a consequence of these damaging processes (24). ROS (reactive oxygen species) then cause a decrease in local antioxidant activity, causing outflow resistance and aggravating the activities of glutathione peroxidase and superoxide dismutase in glaucomatous eyes. An *in vivo* investigation found that IOP elevation contributed to POAG pathogenesis by lowering systemic antioxidant capacity as determined by ferric-reducing activity (25). Additionally, hydrogen peroxide stimulates TM cell rearrangement and affects the integrity of those cells. The combination of TM tissue breakdown in the traditional outflow channel and the neuroinflammation process appears to be the cause of the buildup of ROS and the immune-stimulatory signaling exacerbated by oxidative stress. Decreased antioxidant and increased oxidative are all associated with POAG development.

The current research is distinctive in that it examines the association between dietary antioxidant indices and glaucoma by using data from a population-based survey, which encompasses a broad set of individuals both regionally and demographically. Due to the abundance of additional information provided by the NHANES data, it is possible to account for any confounding variables. And this makes our result more convincing by adopting multivariable logistic regression analysis.

We are aware that there are several shortcomings associated with our study. They are mostly due to the observational and cross-sectional nature of the study methodology. Since it is a cross-sectional research, we are unable to determine whether or not there is a cause-and-effect link between the two variables. In addition to this, there is the risk of confounding with elements that were not included. Besides, self-reported glaucoma may indicate isolated increased intraocular pressure or suspicious optic nerve appearance in the absence of visual field loss. It may potentially reflect many endophenotypes of glaucoma. And self-reported glaucoma may have its own inaccuracy which might have a bias to our results, which needs to be further verified. Additionally, there are even other dietary antioxidants (such as melatonin, saffron, lutein, xanthine, and dietary nitrite intake), which DAI was not taken into account. Finally, the NHANES statistics are not based on a genuine national population but rather on weighted estimates, despite the fact that the weighting method has been well researched and was developed to provide estimates that are claimed to be representative of the population in the United States.

## 5 Conclusion

In conclusion, according to the findings of our research, which was based on data from the 2005–2008 NHANES population, the higher dietary antioxidant indices were related with a decreased risk of glaucoma. However, further large multiple randomized controlled trials are warranted to demonstrate our result.

## Data availability statement

The raw data supporting the conclusions of this article will be made available by the authors, without undue reservation.

## Ethics statement

The studies involving humans were approved by Centers for Disease Control and Prevention. The studies were conducted in accordance with the local legislation and institutional requirements. The participants provided their written informed consent to participate in this study.

## Author contributions

WL: Conceptualization, Data curation, Methodology, Software, Writing – original draft. BW: Supervision, Validation, Writing – review & editing.
